# Role of gender and age in features of Wilson’s disease

**DOI:** 10.3389/fneur.2023.1176946

**Published:** 2023-07-05

**Authors:** Lin Cai, Xiaotao Huang, Yan Ye, Dailan Yang, Linshen Xie, Daigang Fu, Lijun Peng, Dingzi Zhou, Juan Liao

**Affiliations:** ^1^Department of Gastroenterology, West China School of Public Health and West China Fourth Hospital, Sichuan University, Chengdu, China; ^2^Non-communicable Diseases Research Center, West China-PUMC C. C. Chen Institute of Health, Sichuan University, Chengdu, China; ^3^Department of Gastroenterology, 903 Hospital, Jiangyou, China; ^4^Department of Occupational Disease and Toxicosis, West China School of Public Health and West China Fourth Hospital, Sichuan University, Chengdu, China

**Keywords:** Wilson’s disease, gender difference, clinical characteristics, phenotype, age of onset

## Abstract

**Background:**

Wilson’s disease (WD) is a recessive genetic disorder characterized by copper metabolism dysfunction. It is difficult to obtain an accurate diagnosis due to its variable clinical presentation. This study aimed to describe the clinical characteristics and diagnostic particularities in a series of Chinese WD patients.

**Methods:**

The medical records of 371 patients with WD retrieved from January 2005 to December 2020 were retrospectively reviewed.

**Results:**

The incidence of WD has a male predominance in the adult population. However, the difference in sex distribution is not significant in the pediatric population. Females have an earlier symptom onset than males. The most common initial symptoms were neuropsychiatric manifestations both in the pediatric population (49.7%) and adult population (69.8%), and there was a male predominance (61.8%). Eighty-two percent of patients presented with more than two neurologic symptoms. Fifty-two (14%) patients presented with psychiatric symptoms. The most common WD phenotype was the neuropsychiatric form (48%). The age of onset occurred earlier in patients with the hepatic phenotype than in those with the neuropsychiatric phenotype. Moreover, there was a significant difference in sex distribution regarding phenotype. Females presented with a hepatic phenotype more often than males, and the neuropsychiatric phenotype occurred more frequently in males with an older onset age. Further study showed that the age at onset was a deciding factor for predicting the neuropsychiatric phenotype among the hepatic phenotype. However, sex did not correlate with the phenotype.

**Conclusion:**

Males seem to have a higher disease susceptibility, with symptom onset later than females. Males frequently present with a neuropsychiatric phenotype, while females present with a hepatic phenotype. Age at onset was a deciding factor for predicting the WD phenotype. Further studies focusing on the effect of estrogens on the pathology of WD are suggested.

## Introduction

Wilson’s disease (WD) is an inherited autosomal recessive disorder that is caused by pathogenic mutations of ATP7B, which lead to copper metabolism dysfunction. The subsequent gradual aberrant deposition of copper in organs, particularly in the liver and brain, triggers multisystemic symptoms (1). The estimated prevalence of WD is approximately 1/19500, with a higher rate in the Asian and Ashkenazi Jewish populations (2). The clinical presentation and phenotypic characteristics of WD can be highly variable. Patients could present with asymptomatic biochemical abnormalities, hepatitis, steatosis, liver cirrhosis, neurological damage, psychiatric symptoms, and occasionally cardiologic and/or skeletal symptoms. There is no gold standard diagnostic method for establishing the diagnosis of WD. A combination of clinical presentations, abnormal copper metabolism parameters, and/or mutations of the ATP7B gene are necessary to confirm the diagnosis (3).

Wilson’s disease is among a limited number of genetic disorders that can be partly treated or prevented by copper-chelating agents. Early diagnosis and intervention are crucial to minimize the devastating progression of the disease and obtain better outcomes. However, its diagnosis remains a challenge. A delay in diagnosing WD is frequent and may cause irreversible disease deterioration and even fatality. Once the diagnosis is established, lifelong treatment is necessary.

Genetic testing for ATP7B mutations plays an increasing role in the diagnosis of WD. However, the direct molecular genetic diagnosis of WD is difficult at present, as there are more than 500 mutations associated with this condition (4). Additionally, most individuals are compound heterozygotes. Not all suspected patients are able to undergo genetic WD diagnosis or screening, especially those patients in developing countries. The clinical diagnosis mostly relies on both clinical features and biochemical parameters. There is still a lack of reasonably designed clinical trials in China, and most management is based on evidence from other countries. To characterize the patient experience of WD, this study aims to characterize the clinical spectrum and provide a detailed analysis of Chinese WD patients. Moreover, we sought to explore the clinical variants that predict the phenotypes of WD.

## Subjects and methods

Subjects who had a confirmed diagnosis of WD were included. The diagnosis was based on clinical symptoms, abnormal copper metabolism (decreased level of serum ceruloplasmin, and increased 24 h urine copper excretion), the presence of Kayser-Fleischer rings (KF-rings), and ATP7B mutation analysis if available, according to the Leipzig score (5). The demographic, case history, clinical, laboratory, and brain imaging data and the treatment of WD patients were retrospectively collected in a single center from January 2005 to December 2020.

In accordance with a previous study (5), patients were classified into three disease phenotypes as follows: the hepatic form, which presented with signs of liver injury and/or hepatic symptoms; the neuropsychiatric form, which presented with neuropsychiatric symptoms; and the presymptomatic form.

Gender differences have been reported in the course of liver fibrosis (6), neurodegenerative diseases, and WD (7). In this Southwest China population, we investigated the gender differences in the course and clinical features of WD.

Clinical heterogeneity cannot be explained by genetic determinants. To assess whether the clinical variables were associated with phenotype, the following variants were selected for further analysis: age at onset of disease and gender. The ethics committee of West China Fourth Hospital approved this retrospective analysis (HXSY-EC-2021034). The work was carried out in accordance with the code of ethics of the World Medical Association (Declaration of Helsinki).

### Statistical analysis

Statistical analyses were performed with SPSS version 22.0 (IBM, New York, USA). Normally distributed quantitative variables are expressed as the mean ± standard deviation (SD); nonnormally distributed quantitative variables are expressed as the median [IQR]. The relevant parameters were compared using Student’s t test or nonparametric tests, as appropriate. Categorical variables were expressed as counts (percentages) and compared using the χ2 test or Fischer’s exact test when appropriate. A logistic regression analysis was performed to explore the correlation between the clinical parameters and phenotype, and variables with *p* < 0.05 were then used in the multivariate logistic regression analysis. *p-*values lower than 0.05 were considered statistically significant.

## Results

### Patient characteristics

A total of 371 patients who met the criteria for a WD diagnosis were included in the analysis. Demographic and clinical characteristics are summarized in Table 1.

**Table 1:** Demographic, clinical, and laboratory features of Wilson’s disease (WD) patients.

	*n*	Median (range) or %	*p*
Male sex	209	56.3%	0.085
Childhood	87	51.5%	
Adult	122	60.4%	
Age at onset of WD (years)	371	18 (13.5–24)	0.05
Male	209	20 (14–25)	
Female	162	17 (13–23)	
Age at WD diagnosis (years)	371	23 (16–30)	0.336
Male	209	24 (16–30)	
Female	162	21 (16–30)	
Age at initial symptoms present			<0.001^*^
Neuropsychiatric	225	20 (15–25)	
Hepatic	103	16 (9–24)	
Sex distribution of different initial presentations			0.037
Neuropsychiatric involvement	225	60.6%	
Male	139	61.8%	
Female	86	38.2%	
Hepatic involvement	103	27.8%	
Male	51	49.5%	
Female	52	50.5%	
Age distribution in initial presentation			0.002^*^
Neuropsychiatric involvement	225	60.6%	
Childhood	84	49.7%	
Adult	141	69.8%	
Hepatic involvement	103	27.8%	
Childhood	57	33.7%	
Adult	46	22.8%	
Other initial presentation			
Hemorrhage	5	1.3%	
Musculoskeletal	4	1.0%	
Predominant neurological manifestation			
Tremor	132	58.7%	
Dystonia	37	16.4%	
Drooling	14	6.2%	
Dyskinesia	8	3.6%	
Psychiatric symptoms	52	14%	
Cognitive dysfunction	22	42.3%	
Emotional disorders	19	36.5%	
Personality changes	10	19.2%	
Predominant hepatic manifestation			
Increased serum transaminase	34	33.0%	
Abdominal distention	19	18.4%	
Jaundice	11	10.7%	
Positive family history of WD	27	7.2%	
Presence of Kayser-Fleischer ring	241	77.7%	0.101
Male	134	55.6%	
Female	107	44.4%	
Serum ceruloplasmin level (mg/dl)			0.062
Male	169	40 (20–60)	
Female	132	50 (30–80)	
24 h urinary copper excretion (μmol/24 h)			0.46
Male	198	3.99 ± 3.47	
Female	153	3.76 ± 1.63	
Sex distribution of different phenotypes			0.032^*^
Neuropsychiatric form			251
Male	153	61%	
Female	98	39%	
Hepatic form			103
Male	50	48.5%	
Female	53	51.5%	
Treatment			
Sodium dimercaptosulphonate	358	96.2%	
D-penicillamine	10	2.7%	
Zinc salts	41	11%	

WD, Wilson’s disease. ^*^The patients with hepatic injury as the age at initial symptoms were significantly younger than that of patients presenting with neuropsychiatric symptoms. ^*^A higher proportion of the adult population initially presented with neuropsychiatric symptoms than that in the pediatric population.

Regarding the age of onset, 169 (45.6%) subjects were in the pediatric age group (<18 years of age), and 202 (54.4%) were in the adult group. A total of 87.8% of patients were 5 to 35 years of age, and 5.7% were over 40 years of age. The minimum age of onset was 3 years, and the maximum was 54 years. There was a male predominance in adults, with 122 patients (60.4%) being male. In the pediatric population, the gender difference was not obvious, with only 87 (51.5%) being male. However, there was no significant difference in sex distribution between the adult and pediatric populations (*p* = 0.085). Of the patients studied, 27 patients (7.2%) had a family history of WD, with 26 siblings detected by family screening.

### Gender profile in the onset and diagnosis of disease

Regarding the onset of disease, females had an earlier symptom onset than males (Table 1) (*p* = 0.050). The diagnosis of WD was made at a median age of 23 (range 16–30) years, and there was no statistically significant difference in sex distribution (*p* = 0.336).

The initial symptoms were neuropsychiatric abnormalities in 225 patients and hepatic abnormalities in 103 patients. The patients with hepatic injury as the initial symptom were 16 (9–24) years of age, which is significantly younger than that of patients presenting with neuropsychiatric symptoms (*p* < 0.001). Although neuropsychiatric symptoms were also commonly present in childhood (49.7%), a higher proportion of the adult population initially presented with neuropsychiatric symptoms than that in the pediatric population (*p* = 0.002). Further analyses suggested that a male predominance was observed in patients with neuropsychiatric symptoms compared with those with hepatic symptoms as initial symptoms (*p* = 0.037). In the hepatic injury group, the sex distribution was nearly equal.

The most common neurological manifestations were tremor (58.7%) and dysarthria (16.4%). In addition, 82% of patients presented with more than two neurologic symptoms. Moreover, 52 (14%) patients presented with psychiatric symptoms. The common manifestations were cognitive dysfunction (42.3%), emotional disorders (36.5%), and personality changes (19.2%). The main hepatic features were increases in the levels of aminotransferases (33.0%) and the incidences of abdominal distention (18.4%) and jaundice (10.7%).

### Gender profile in other clinical features

A total of 241 (77.7%) patients presented with KF-rings in the cornea. Although there was no statistically significant difference in the sex distribution (*p* = 0.101), a male predominance (55.6%) was observed in patients presenting with KF-rings (Table 1).

At presentation, 3.3% of patients had normal serum ceruloplasmin levels. Although there were no significant differences, males had higher 24 h urine copper excretion (*p* = 0.46) and serum ceruloplasmin levels (*p* = 0.062) than females.

### Gender profile in WD phenotype

At presentation, 251 (67.7%) patients had the neuropsychiatric phenotype, and 103 (27.8%) patients had the hepatic phenotype of the disease (Table 1). The differences in the sex distribution between the neuropsychiatric phenotype and the hepatic phenotype were statistically significant (*p* = 0.032). Females presented more often with a hepatic phenotype than males. Neuropsychiatric presentation occurred more frequently in males. Females had a significantly earlier age of onset than males in the neuropsychiatric phenotype (*p* = 0.003, Table 2). There were no significant differences in the gender profile regarding the presence of KF-rings, serum ceruloplasmin concentration, and 24-h urinary copper excretion in different WD phenotypes.

**Table 2:** The characteristics of different Wilson’s disease phenotypes in females and males.

	Females	Males	*p*
Neuropsychiatric form			
Age at onset (years)	18 (13–23)	21 (16–25)	0.003^*^
Presence of KF-ring	83 (84.7%)	112 (74.7%)	0.06
Serum ceruloplasmin level	40 (20–70)	40 (20–60)	0.50
24 h urinary copper	3.90 (2.83–4.90)	3.7 (2.54–4.96)	0.552
Hepatic form			
Age at onset (years)	17 (9–24)	14 (7.75–23.25)	0.279
Presence of KF-ring	19 (70.4%)	19 (76%)	0.647
Serum ceruloplasmin level	50 (30–90)	50 (20–70)	0.67
24 h urinary copper	3.20 (2.23–4.90)	3.33 (2.49–4.70)	0.792
Presymptomatic form			
Presence of KF-ring	5 (100%) 3 (75%)	0.236	
Serum ceruloplasmin level	50 (30–70)	45 (32.5–165)	0.66
24 h urinary copper^#^	3.54 ± 1.41	3.19 ± 1.11	0.61

^*^The difference has statistical significance. ^#^Only the 24 h urinary copper in the presymptomatic phenotype was normally distributed and is presented as the mean ± standard deviation. Others are presented as the median (IQR).

For disease phenotype prediction, both univariate and multivariate logistic regression were performed. As shown in Table 3, only age at onset was a deciding factor when predicting hepatic phenotype and neuropsychiatric phenotype. Early onset age is inclined toward the hepatic phenotype rather than the neuropsychiatric phenotype. There was no significant correlation between sex distribution and the WD phenotype in multivariate logistic regression.

**Table 3:** Univariate and multivariate logistic regression of factors associated with the neuropsychiatric phenotype and hepatic phenotype.

Neuropsychiatric form^a^	Univariate		Multivariate	
	OR	*p*	OR	*p*
Sex (female)	0.604	0.033	0.629	0.053
Age at onset(years)	1.043	0.002	1.041	0.003

^a^The reference category is the hepatic form.

## Discussion

There are five main findings in our study. ① There was a male predominance in the prevalence of WD in the adult population. However, the gender difference was not significant in childhood. ② Females have an earlier symptom onset than males, but there is no significant gender difference regarding the age of diagnosis. ③ The most common initial symptoms were neuropsychiatric manifestations, with a significant male predominance. The tendency becomes more apparent in adults. Hepatic injury is present at an earlier age of onset than neuropsychiatric symptoms. ④ The most common WD phenotype was the neuropsychiatric form. There was a significant gender difference. Females presented more often with a hepatic phenotype than males. Neuropsychiatric presentation occurred more frequently in males. ⑤ Earlier age at onset was a deciding factor for predicting the hepatic phenotype rather than the neuropsychiatric phenotype.

WD is a highly heterogeneous disease. There is still a lack of sufficient knowledge on the natural history of untreated patients with WD. It is well known that WD trends toward a young age of onset, as most diagnoses are made between the ages of 5 and 35 years. Our results demonstrated that the incidence of age distribution in the population was very similar to that in a previous report (8). In our study, there was a male predominance in the prevalence of this condition in adults. Interestingly, no significant gender difference was observed in childhood. These findings indicate that sex hormones may play a role in the pathogenesis of WD, with estrogens exerting antioxidant, neurotrophic, and anti-inflammatory effects (9).

There was a gender difference regarding disease presentation in WD patients. In accordance with a previous study (10), the neurologic manifestations start later and are more common in males. Hepatic presentation was more common among females. A possible explanation is that estrogens exert a protective role in the brain. Gromadzka et al. demonstrated that iron metabolism differences may be associated with sex-related clinical heterogeneity (11). Further studies are needed to elucidate the underlying mechanisms.

Interestingly, the occurrence of neurological symptoms in childhood is higher than that in in previous studies (12). The discrepancies could possibly be explained by the median age of children in our study being older than 10 years. Neurological symptoms are more prevalent in older children (13).

Psychiatric symptoms are nonspecific, and the clinical features are variable. That may cause difficulties in diagnosis. In this study, the occurrence of psychiatric manifestations was lower than that in a previous study (14), and a male predominance was observed. In fact, neuropsychiatric WD is less well appreciated in childhood. Unfortunately, there is still a lack of dedicated scales to assess the psychiatric symptoms of WD. In this study, the diagnosis of the psychiatric form of WD is based on psychiatric signs and symptoms, psychiatric examination. It is difficult to assess whether psychiatric symptoms occurred as a result of central nervous system involvement since psychiatric symptoms may not occur together with neurologic symptoms. Further studies are needed to explore this question in newly diagnosed cases with psychopathologic assessment. There is also a need to elaborate a validate evaluation scale, with a multidisciplinary team involving hepatologists, neurologists, and psychiatrists to assess the psychiatric profile of WD.

In our study, both hepatic and neurological WD may initially display subtle symptoms that are easily overlooked. Thus, diagnostic vigilance should be proposed when faced with any liver disease and/or neurological disease with unknown etiological factors. Moreover, careful history-taking and review of laboratory data and imaging are necessary.

ATP7B gene mutations are the pathophysiological basis of WD (15), but the correlation between genotype and disease phenotype still lacks evidence. Ferenci et al. demonstrated no correlations between genotype and phenotype regarding initial symptomatic manifestation (16). Both siblings and monozygotic twins commonly have different WD phenotypes, and the same gene mutations display clinical variability among isolated populations (17–19). Moreover, several reports describe that clinical phenotypes are influenced by age (20) and sex (16). In our study, only the age of onset was a deciding factor for distinguishing the neuropsychiatric phenotype from the hepatic phenotype. Further studies are needed to explore the specific role of epigenetic factors and sex hormones and their mechanisms.

The length of this study, including the that of the follow-up, is insufficient. Second, we did not measure the level of estrogen. Third, due to the retrospective design of this study, the mechanisms underlying the sex differences remain unclear.

## Conclusion

In summary, the data obtained in a cohort of 371 patients with WD clearly show gender differences in the clinical features of WD, and the age of onset was a deciding factor for disease phenotype prediction.

## Data availability statement

The raw data supporting the conclusions of this article will be made available by the authors, without undue reservation.

## Ethics statement

The studies involving human participants were reviewed and approved by the Ethics Committee of West China Fourth Hospital. Written informed consent from the participants was not required to participate in this study in accordance with the national legislation and the institutional requirements.

## Author contributions

LC and XH implemented the data analysis, interpreted the results, and drafted this manuscript. JL provided critical revision of the manuscript for important intellectual content. LC, YY, and XH did the preliminary analysis. DY collated the results. LX, DF, LP, DZ, and JL revised the manuscript. All authors contributed to the conception and design of the research and read and approved the final manuscript.

## Conflict of interest

The authors declare that the research was conducted in the absence of any commercial or financial relationships that could be construed as a potential conflict of interest.

The handling editor HS declared a shared parent affiliation with the author's LC, YY, DY, LX, DF, LP, DZ, and JL at the time of review.

## Publisher’s note

All claims expressed in this article are solely those of the authors and do not necessarily represent those of their affiliated organizations, or those of the publisher, the editors and the reviewers. Any product that may be evaluated in this article, or claim that may be made by its manufacturer, is not guaranteed or endorsed by the publisher.
